# Supplementary material for: Multiplexed screening reveals how cancer-specific alternative polyadenylation shapes tumor growth in vivo
Source: Nat Commun. 2024 Feb 1;15:959. doi: 10.1038/s41467-024-44931-x (PMC10834521; doi:10.1038/s41467-024-44931-x)

Supplementary Information

SUPPLEMENTAL FIGURE 1

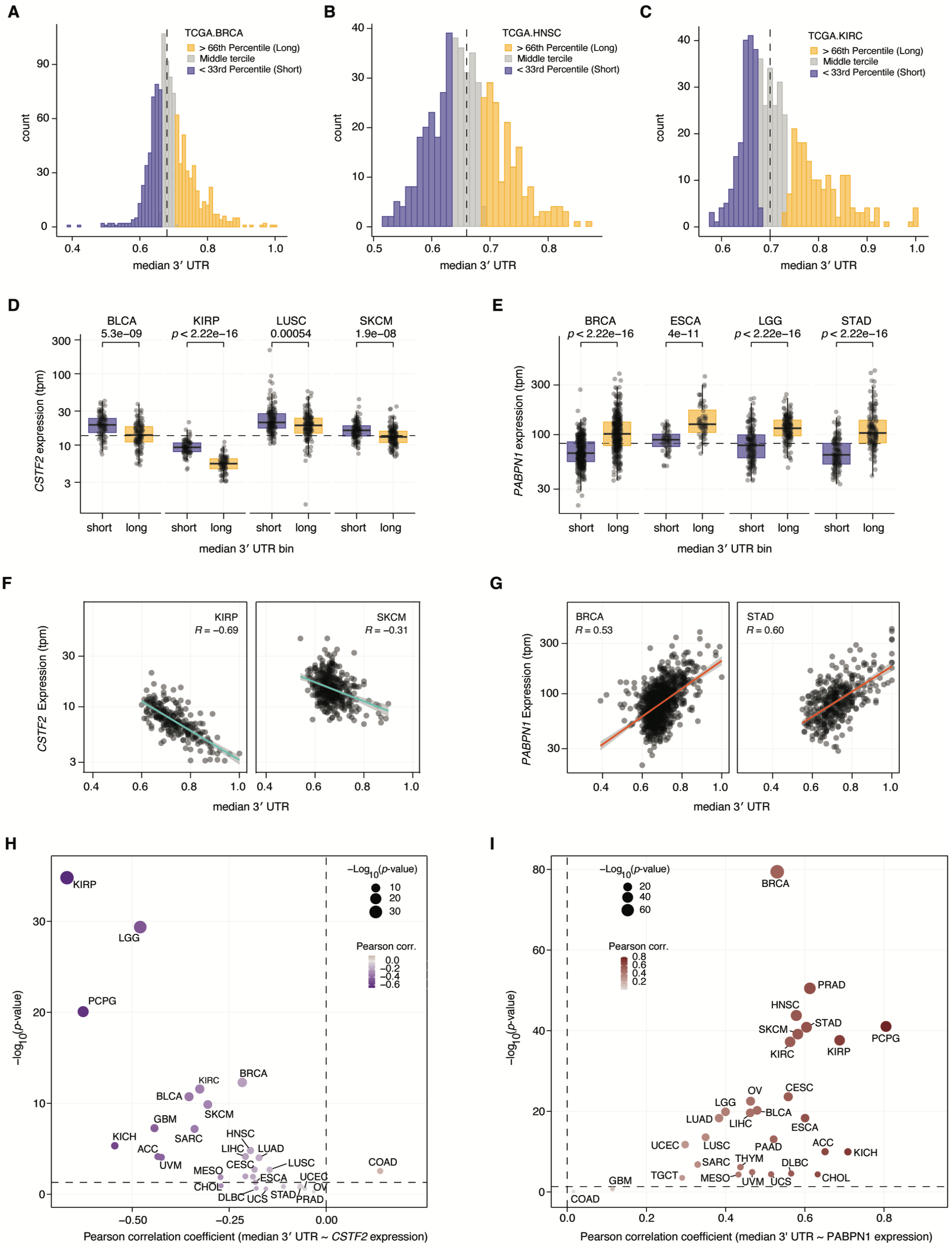

Supplemental Figure 1. Median 3′ UTR correlates with known global regulators of 3′ UTR length.

(**A-C**) Distributions and binning of median 3′ UTR length for invasive breast carcinoma (A) (n = 730 RNA-seq samples), head and neck squamous cell carcinoma (B) (n = 374 RNA-seq samples), and kidney renal cell carcinoma (C) (n = 374 RNA-seq samples), calculated using the DaPars algorithm to quantify gene-level 3′ UTR lengths per sample (15).
(**D**) Box plot comparing *CSTF2* expression (TPM) per median 3′ UTR bin for four TCGA cancer subtypes, bladder urothelial carcinoma (BLCA), kidney renal papillary cell carcinoma (KIRP), lung squamous cells carcinoma (LUSC), and cutaneous melanoma (SKCM). *P* values calculated with a two-sided Wilcoxon rank-sum test.
(**E**) Box plot comparing *PABPN1* expression (TPM) per median 3′ UTR bin for four TCGA cancer subtypes, invasive breast carcinoma (BRCA), esophageal carcinoma (ESCA), brain lower grade glioma (LGG), and stomach adenocarcinoma (STAD). *P* values calculated with a two-sided Wilcoxon rank-sum test.
(**F**) Scatter plots of median 3′ UTR length versus *CSTF2* expression (TPM) per sample for two TCGA cancer subtypes, kidney renal papillary cell carcinoma (KIRP) and cutaneous melanoma (SKCM). *R* values from Pearson correlation.
(**G**) Scatter plots of median 3′ UTR length versus *PABPN1* expression (TPM) per sample for two TCGA cancer subtypes, invasive breast carcinoma (BRCA) and stomach adenocarcinoma (STAD). *R* values from Pearson correlation.
(**H**) Volcano plot of the Pearson correlation per TCGA cancer subtype plotted against the -$\log_{10}$(*p* value) of that Pearson correlation coefficient for median 3′ UTR length versus *CSTF2* expression (TPM).
(**I**) Volcano plot of the Pearson correlation per TCGA cancer subtype plotted against the -$\log_{10}$(*p* value) of that Pearson correlation coefficient for median 3′ UTR length versus *PABPN1* expression (TPM).

SUPPLEMENTAL FIGURE 2

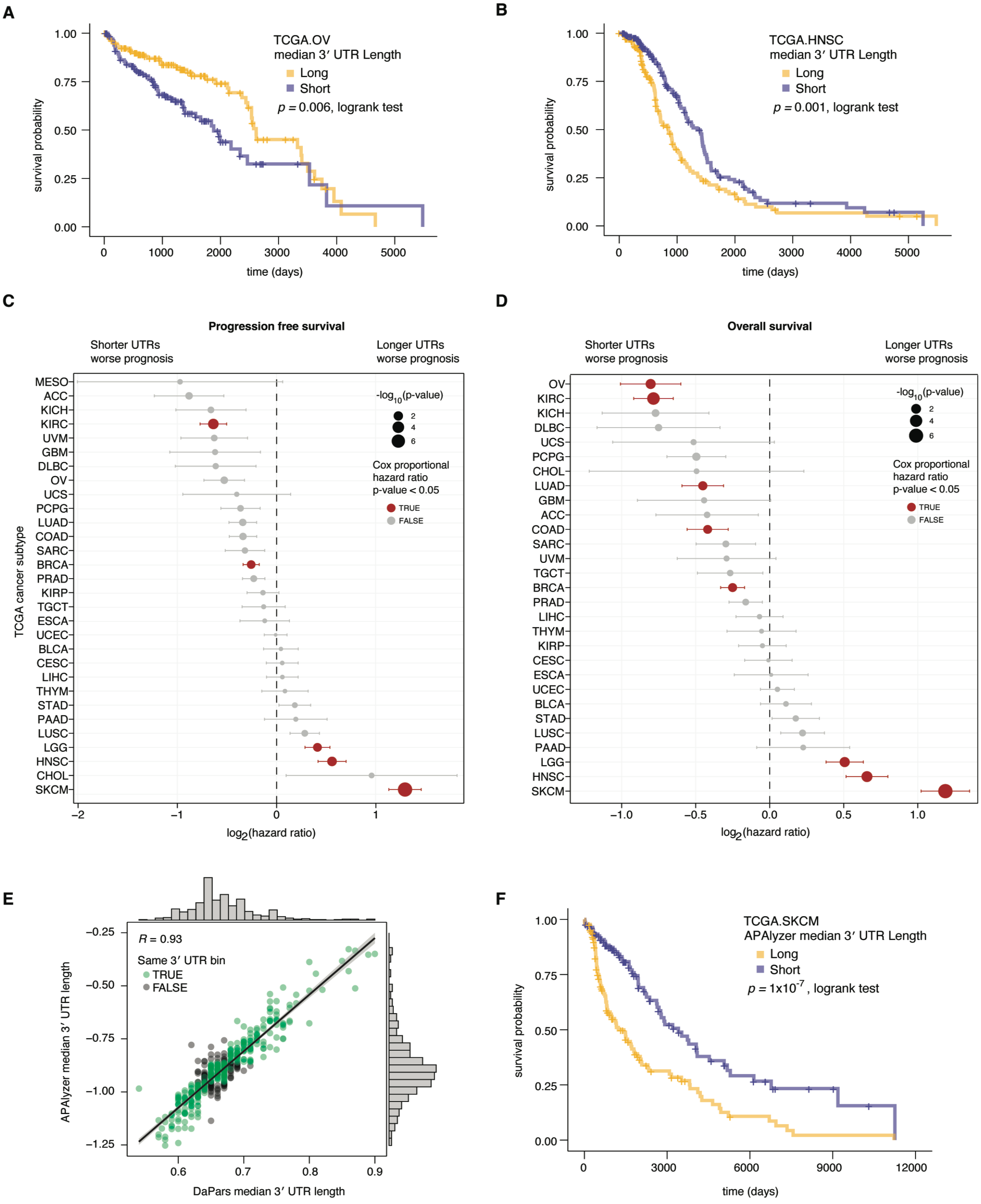

Supplemental Figure 2. Global median 3′ UTR length correlates with patient outcomes in several cancer subtypes.

(**A**) Kaplan-Meier analysis comparing overall survival of TCGA ovarian serous cystadenocarcinoma (OV) samples binned as short or long median 3′ UTR samples. *P* values from a two-sided logrank test.
(**B**) Kaplan-Meier analysis comparing overall survival of TCGA head and neck squamous cell carcinoma (HNSC) samples binned as short or long median 3′ UTR samples. *P* values from a two-sided logrank test.
(**C-D**) Forest plot of the $\log_2$(hazard ratio) per TCGA cancer subtype comparing progression-free survival (C) or overall survival (D) for long versus short median 3′ UTR bin. Point sizes are scaled to $-\log_{10}$(*p* value) of the hazard ratio per subtype.
(**E**) Scatter plot comparing median 3′ UTR length calculated using DaPars (15) or APAlyzer (23) computational pipelines, *R* from Pearson correlation. Points are colored by if they are stratified into the same bin (short, medium or long) by both computational algorithms, where green indicates they are classified similarly by both algorithms and black indicates they are not.
(**F**) Kaplan-Meier analysis comparing overall survival of cutaneous melanoma samples with shorter versus longer median 3′ UTRs calculated using APAlyzer, *P* values from a two-sided logrank test.

SUPPLEMENTAL FIGURE 3

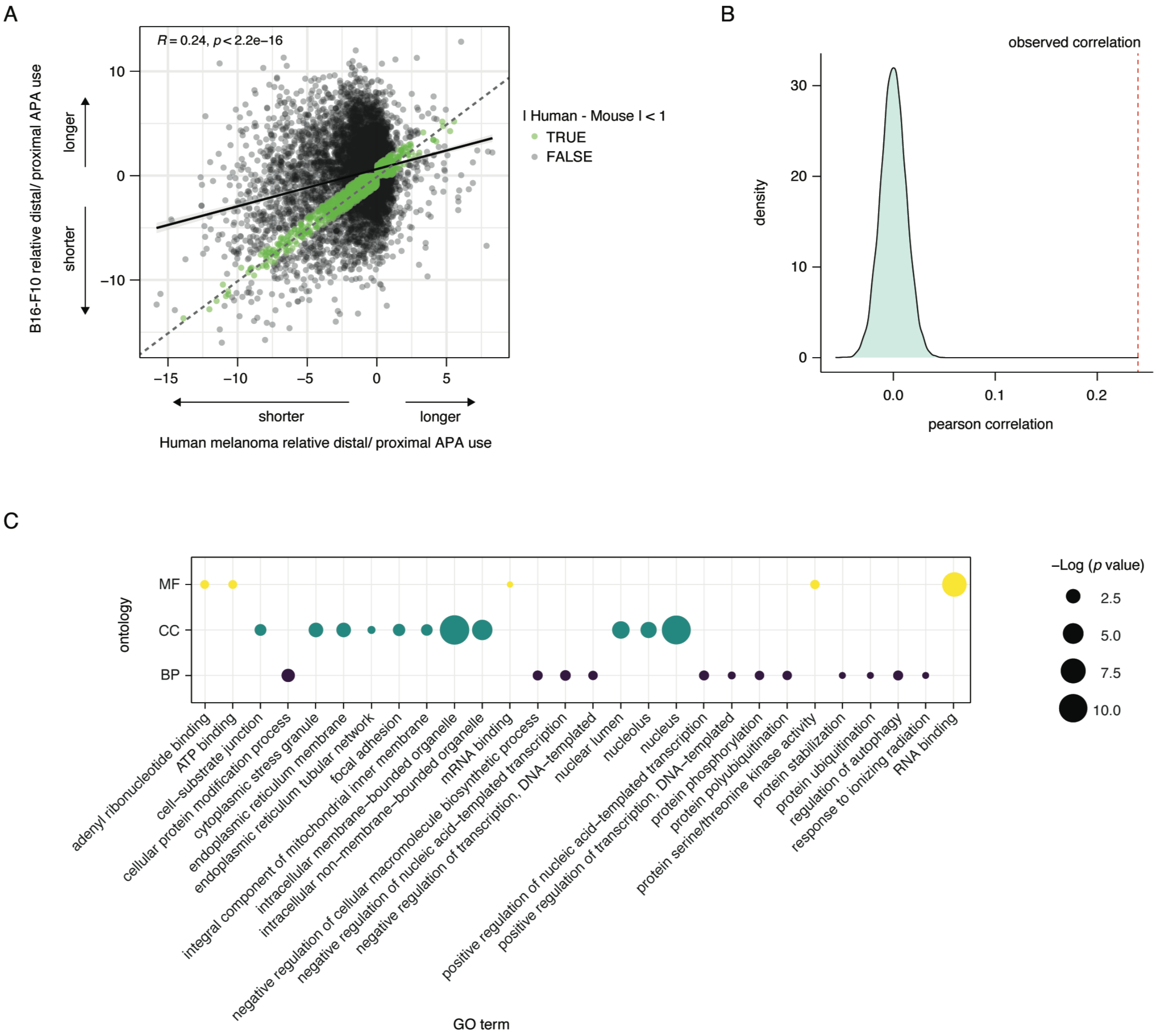

Supplemental Figure 3. APA in human and mouse melanoma are correlated.

(**A**) Gene level 3′ UTR lengths correlated between human clinical melanoma samples and B16-F10 mouse melanoma cells both calculated using APAlyzer, each value reflects the log2 (distal reads / proximal reads) for that given gene ortholog in mouse and human. *R* and *P* value calculated from Pearson correlation. Dotted line indicates x = y and points colored in green are genes where the absolute difference between the mouse and human values are less than 1, indicating the 3′ UTR lengths are similar in mouse and human.
(**B**) Distribution of Pearson coefficients obtained from 10,000 random pairings of gene level 3′ UTR lengths correlated between Human clinical melanoma samples and B16-F10 mouse melanoma cells (data from panel A), dashed red line indicates the observed *R* from Pearson correlation of the actual data.
(**C**) GO analysis of genes where the 3′ UTR lengths are highly correlated between human and mouse melanoma (indicated in green in panel A).

SUPPLEMENTAL FIGURE 4

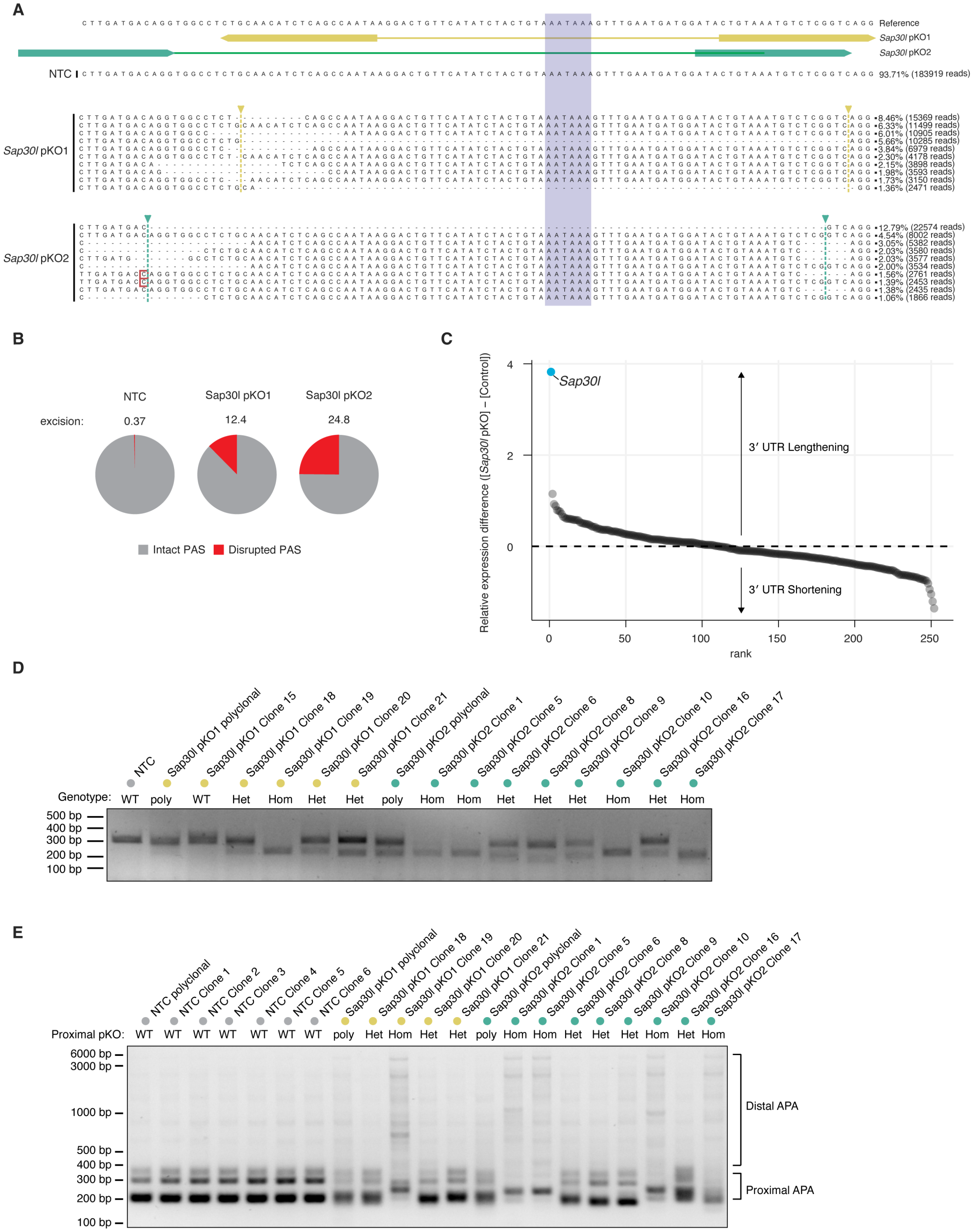

Supplemental Figure 4. CRISPR-Cas9 paired-guide RNAs can be used to delete polyadenylation signals.

(**A**) Schematic of *Sap30l* proximal poly(A) sequence targeted pgRNAs, *Sap30l* pKO1 and pKO2. Next-generation sequencing results showing the major detected alleles from three polyclonal B16-F10 Cas9-expressing cells treated with either a non-targeting control (NTC), *Sap30l* pKO1 or *Sap30l* pKO2 pgRNA as indicated and submitted for next-generation sequencing of the target locus (n = 1 NGS run per genotype).
(**B**) Pie chart demonstrating the fraction of next-generation sequencing reads with an intact or disrupted polyadenylation signal sequence for each cell line treated with the indicated pgRNA.
(**C**) Waterfall plot of Poly(A)-seq analysis comparing polyclonal B16-F10 Cas9-expressing cells treated with either a control (N=2) or *Sap30l* pKO pgRNA (N=4). Relative expression difference is calculated using APAlyzer (23) and is defined as the difference in the $\log_2$ ratio of (distal/proximal) per transcript between *Sap30l* pKO treated and control treated B16-F10 Cas9-expressing cells.
(**D**) Genotyping PCR of genomic DNA obtained from the indicated polyclonal or monoclonal or monoclonal cell line confirming single or dual KO of the proximal poly(A) signal sequence (lower band indicates excision) and genotypes indicated as wild-type (WT), heterozygous excision (Het) or homozygous excision (Hom).
(**E**) Nested RT-PCR of the *Sap30l* 3′ UTR in polyclonal and monoclonal B16-F10 Cas9-expressing cell lines treated with a non-targeting (NTC) pgRNA, *Sap30l* pKO1 or *Sap30l* pKO2. Proximal poly(A) signal presence is noted as proximal pKO, where WT = two wild type poly(A) signals, Het = single excision of poly(A) signal and Hom = double excision of poly(A) signal. Monoclonal lines are treated as independent biological replicates (n = 6 WT, n = 4 Sap30l pKO1, and n = 8 Sap30l pKO2).

SUPPLEMENTAL FIGURE 5

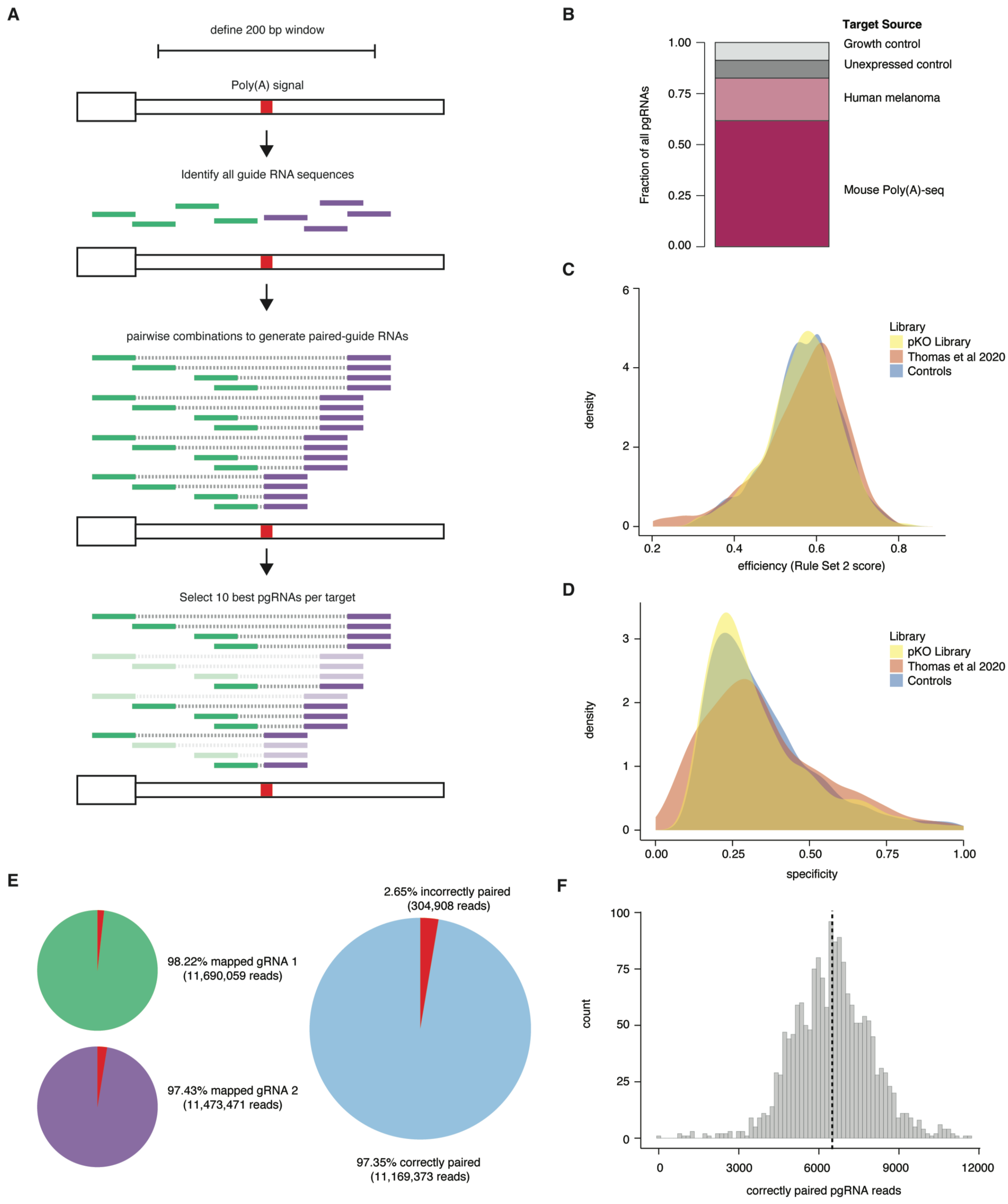

Supplemental Figure 5. Rational design of a CRISPR-Cas9 paired-guide RNA library to target polyadenylation signals.

(**A**) Diagram of computational steps taken to generate a CRISPR-Cas9 paired-guide RNA library to delete proximal polyadenylation signal sequences of interest.
(**B**) Library divided into the fraction of total pgRNAs which were selected based on how the specific target was selected to be included, referred to as the target source (n = 1718 pgRNAs).
(**C**) Distribution of on-target efficiency, using Rule Set 2 scores (53) per pgRNA for the pgRNAs targeting proximal poly(A) sites (pKO Library) compared to a previously published pgRNA library (29) or the 150 pgRNAs targeting poly(A) sites in unexpressed genes (Controls), which were randomly sampled from all possible control pgRNAs to match the distribution of on and off-target scores of the pKO library.
(**D**) Distribution of target specificity per pgRNA for the pgRNAs targeting proximal poly(A) sites (pKO Library) compared to a previously published pgRNA library (29) or the pgRNAs targeting poly(A) sites in unexpressed controls.
(**E**) Next-generation sequencing of the final plasmid pool of the cloned proximal poly(A) site KO library. The number of reads and the fraction of those reads correctly mapping to gRNA1, gRNA2 and then the correct pairing of those gRNAs together are shown.
(**F**) Histogram of the number of reads with the correct gRNA mapping and pairing per each of the 1718 pgRNAs included in the final library. Dotted line indicates median number of reads across all pgRNAs.

SUPPLEMENTAL FIGURE 6

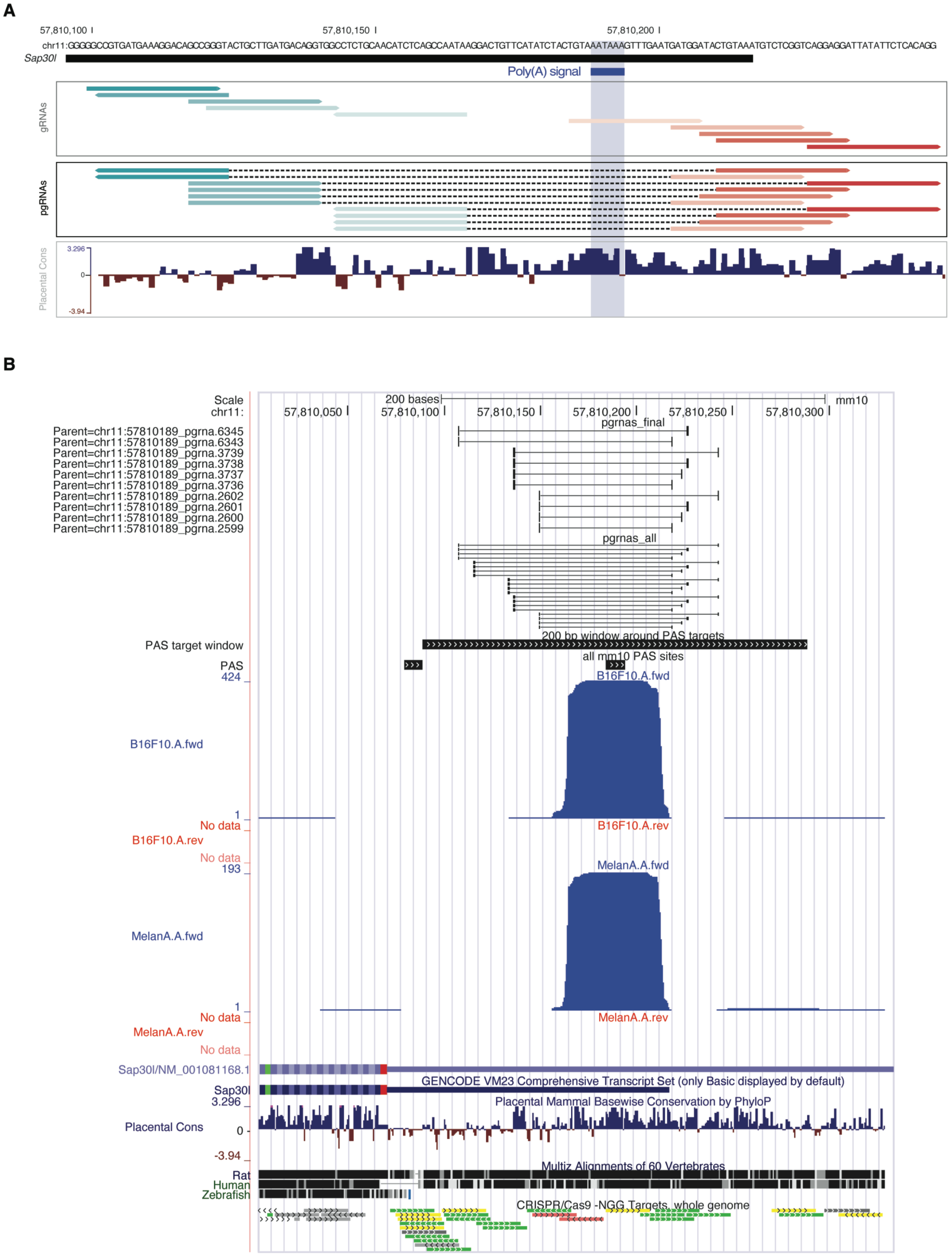

Supplemental Figure 6. Visualization of proximal poly(A) knock out library.

(**A**) *Sap30l* 3′ UTR DNA sequence with the annotated proximal poly(A) signal sequence, individual gRNAs identified within a 200 base-pair window centered on the poly(A) signal sequence, the 10 selected final pgRNAs designed to delete the proximal poly(A) site and the genetic conservation per base-pair across placental mammals.
(**B**) *Sap30l* 3′ UTR DNA sequence in the UCSC Genome Browser window. The session contains several tracks including annotated poly(A) signal sites (PAS), all pgRNAs generated within the 200 base-pair window per poly(A) site and the final selected 10 pgRNAs. There are also 4 tracks of Poly(A)- seq mapped in a stranded fashion from Melan-A and B16-F10 cells (a forward and reverse strand track for each cell line).

SUPPLEMENTAL FIGURE 7

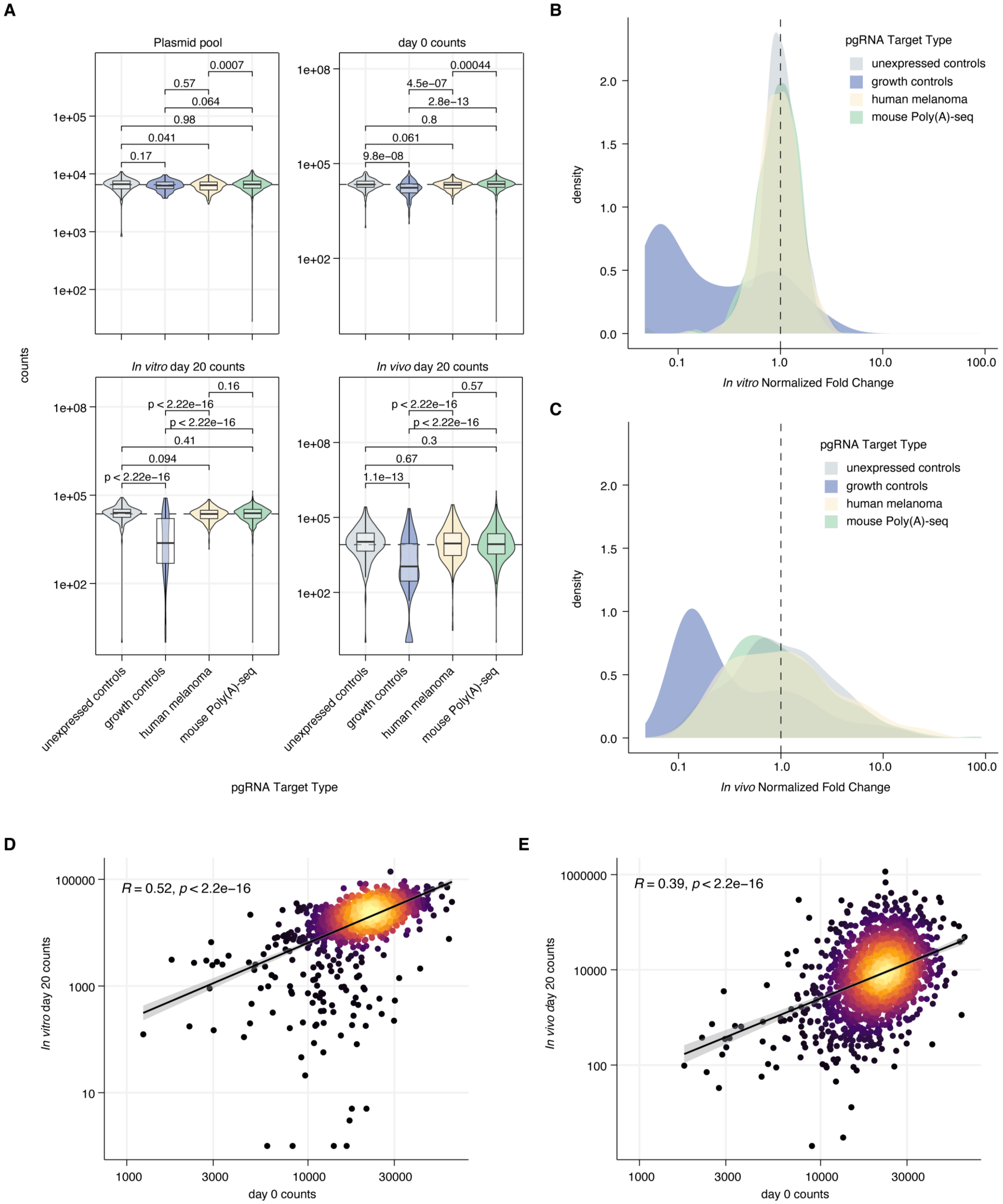

Supplemental Figure 7. Performance of CRISPR-Cas9 pgRNA screening platform.

(**A**) Violin plots of all raw counts per each pgRNA at each experimental time point (plasmid pool, day 0, *in vitro* day 20 or *in vivo* day 20) separated by the target type. *P* values from two-sided Wilcoxon rank-sum tests. Pooled data from n = 8 biological replicates.
(**B**) Density plot of the *in vitro* fold-change of each pgRNA separated by target type, all normalized to the median of 150 pgRNAs targeting poly(A) sites in unexpressed genes. Dotted line at the normalized median of the control pgRNAs which was set to 1. Pooled data from n = 8 biological replicates.
(**C**) Density plot of the *in vivo* fold-change of each pgRNA separated by target type, all normalized to the median of 150 pgRNAs targeting poly(A) sites in unexpressed genes. Dotted line at the normalized median of the control pgRNAs which was set to 1. Pooled data from n = 8 biological replicates.
(**D**) Scatter plot of day 0 pgRNA reads versus *in vitro* day 20 pgRNA reads (summed counts across n = 8 replicates), *R* and *P* value from Pearson correlation.
(**E**) Scatter plot of day 0 pgRNA reads versus *in vivo* day 20 pgRNA reads (summed counts across n = 8 replicates), *R* and *P* value from Pearson correlation.

SUPPLEMENTAL FIGURE 8

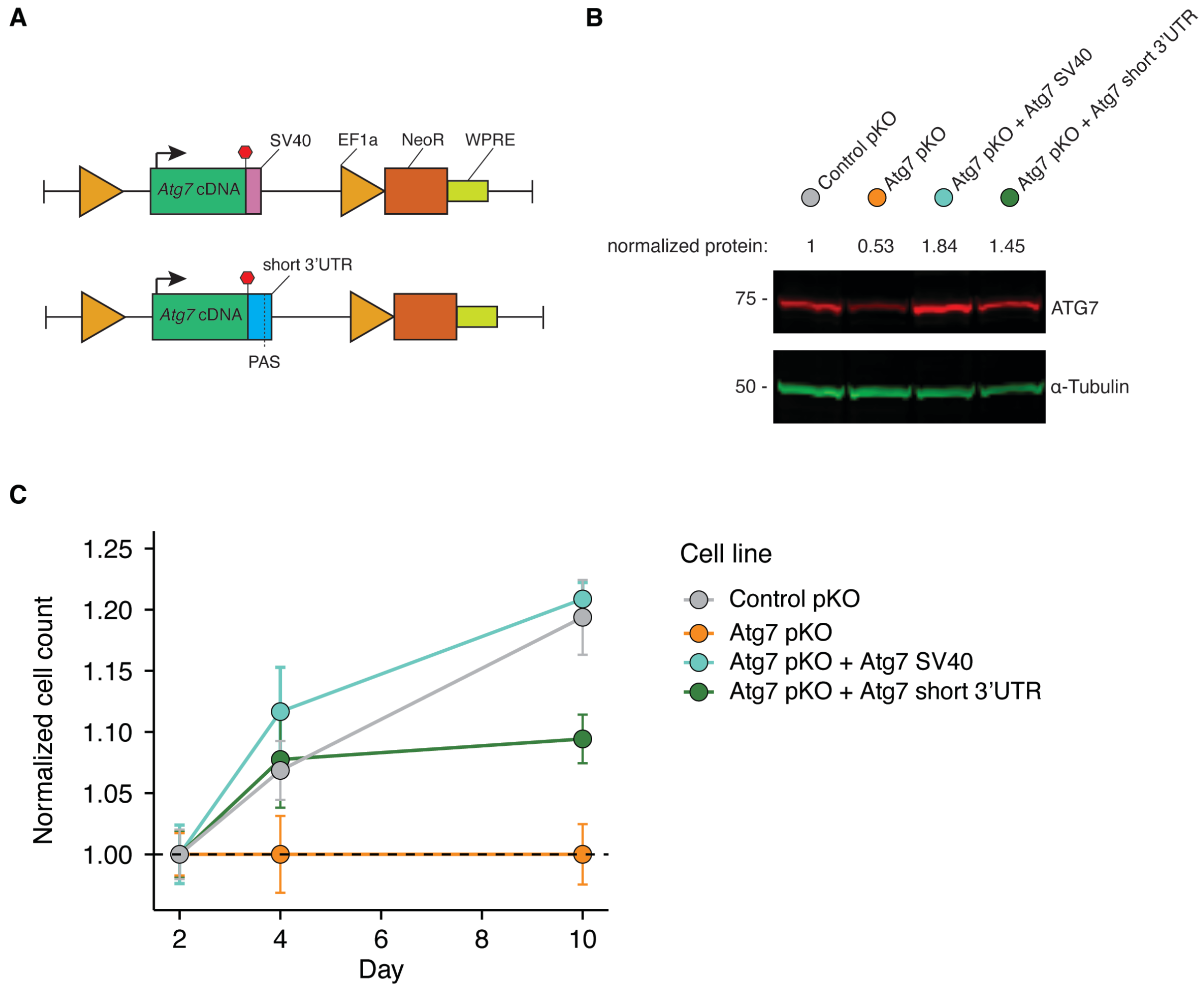

Supplemental Figure 8. Transgenic restoration of ATG7 protein rescues the *Atg7* pKO growth phenotype.

(**A**) Schematic of two *Atg7* cDNA constructs harboring identical coding sequences, but distinct 3′ UTRs, with either an SV40 poly(A) signal or the endogenous 3′ UTR and the proximal poly(A) site.
(**B**) Immunoblot of protein collected from Cas9-expressing B16-F10 cells treated with either a control pgRNA or *Atg7* pKO pgRNA with stable expression of the indicated *Atg7* cDNA construct. Protein ratio normalized to a-Tubulin concentration and then to the control pKO protein ratio. Representative blot from n = 2 technical replicates.
(**C**) *In vitro* cell growth of Cas9-expressing B16-F10 cells treated with a control pgRNA or *Atg7* pKO pgRNA with the indicated cDNA constructs as measured by CellTiter-Glo. Measurement is the average of n = 3 technical replicates +/- standard error of the mean.

SUPPLEMENTAL FIGURE 9

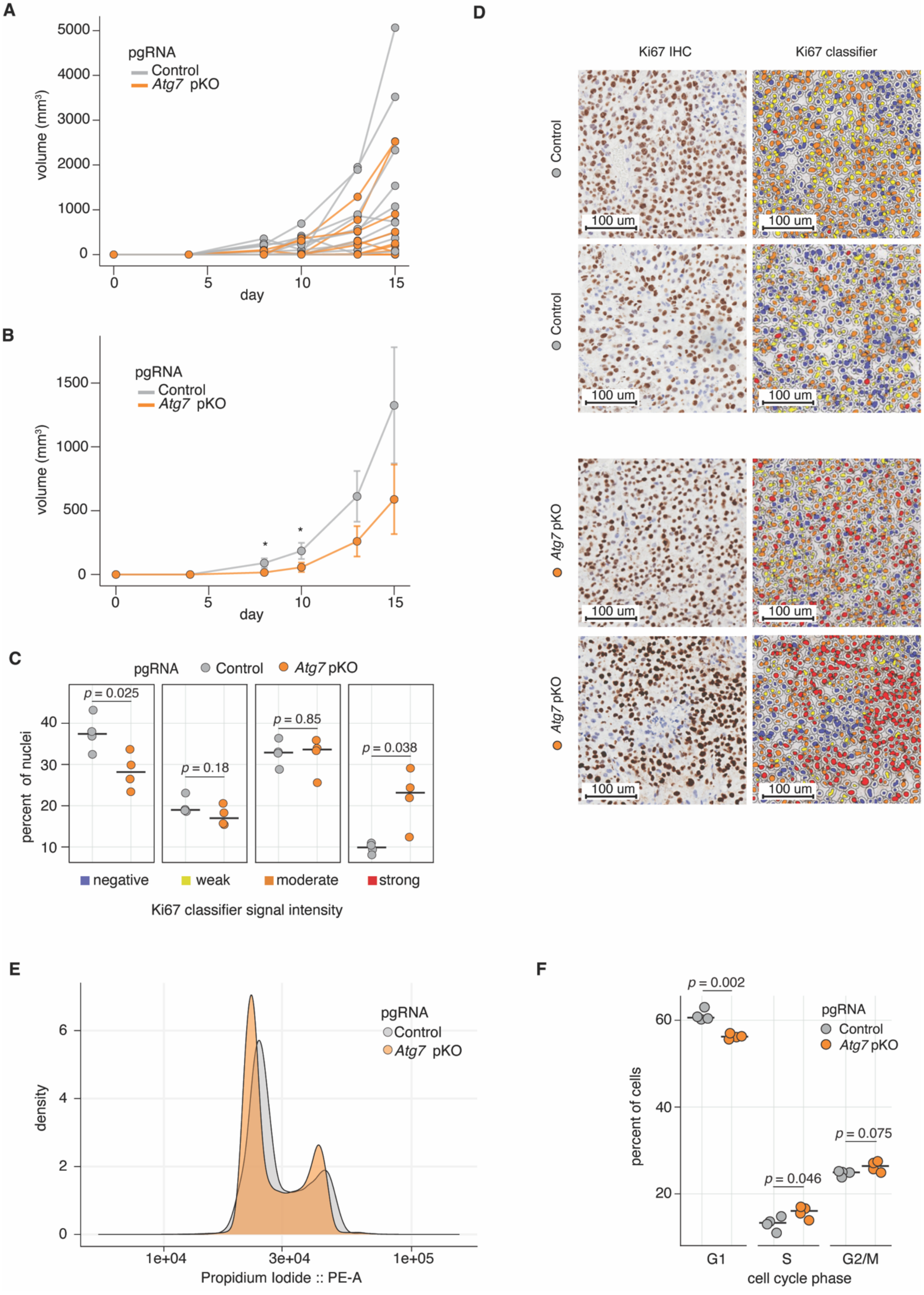

Supplemental Figure 9. *Atg7* 3′ UTR lengthening slows tumor growth in an immunocompetent host.

(**A**) Spider plot showing measured B16-F10 tumor volume over time for cells treated with a control paired-guide RNA (grey) or a paired-guide RNA targeting the proximal poly(A) site in ATG7 (orange) (n = 12 tumors per genotype).
(**B**) Median tumor volume ± S.E.M. per genotype plotted over time (n = 12 tumors per genotype). Significance denoted as $*p < 0.05$, $**p < 0.01$ or $***p < 0.001$ using a two-sided Student's *t*-test (exact *p* values 1, 1, 0.038, 0.049, 0.073 and 0.091).
(**C**) Quantification of percent of nuclei quantified as negative, weak, moderate or strong staining for Ki67 signal (n = 4 images from 4 distinct tumors per genotype, biological replicates). For each image, the entire slide is processed, only excluding areas if they are easily discernible as non-tumor tissue. Significance calculated with a two-sided Student's *t*-test.
(**D**) Additional representative images of raw Ki67 IHC signal and mask of nuclei classified as negative (blue), weak (yellow), moderate (orange) or strong (red) staining nuclear signal. Each row is reflective of a distinct tumor.
(**E**) Histogram of pooled propidium iodide stained B16-F10 cells treated with the indicated paired-guide RNA. Density plots are reflected events gated for live, single cells
(**F**) B16-F10 Cas9-expressing cells treated with a control or *Atg7* pKO pgRNA cells stained with propidium iodide and then analyzed using Dean-Jett-Fox classification for cell cycle stage from FlowJo v10. Quantification of cell cycle stage from propidium iodide staining and flow cytometry (n = 4 samples per genotype). Significance calculated with a two-sided Student's *t*-test.

SUPPLEMENTAL FIGURE 10

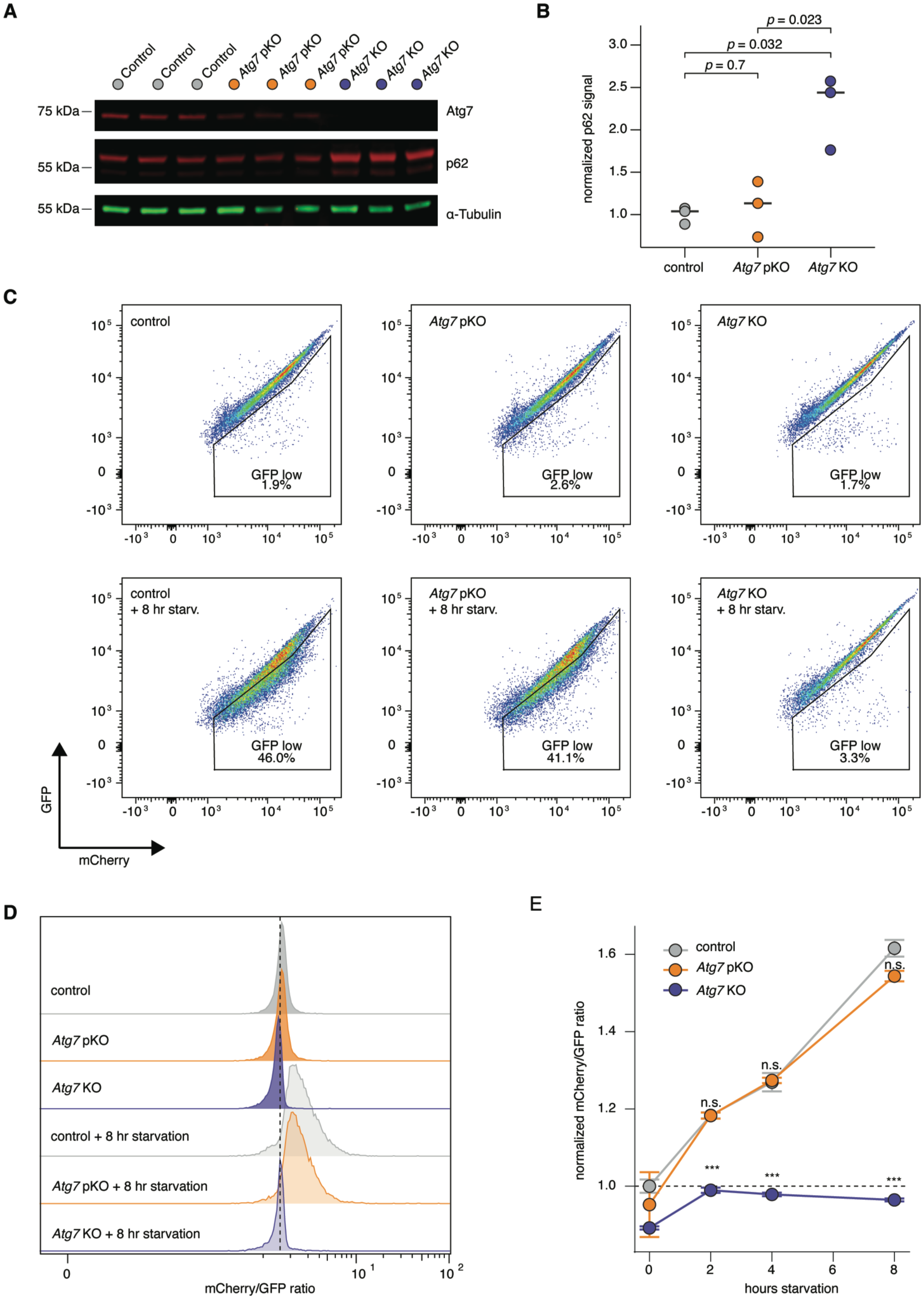

Supplemental Figure 10. Melanoma cells with lengthened *Atg7* 3′ UTRs display no obvious autophagy deficits.

(**A**) Immunoblot of protein collected from B16-F10 Cas9-expressing cells treated with either a control, *Atg7* pKO or *Atg7* gene KO pgRNA completed in technical triplicate. Western blot is stained for Atg7 and p62/Sqstm1, as well as alpha-tubulin as a loading control.
(**B**) Quantification of p62/Sqstm1 protein levels per genotype, normalized to loading control and then normalized to the median of B16-F10 Cas9-expressing cells treated with a control pgRNA. *P* values from a two-sided Student's *t*-test.
(**C**) B16-F10 Cas9-expressing cells treated with control, *Atg7* pKO or *Atg7* gene KO pgRNAs were stably integrated with a LC3-mCherry-GFP autophagy reporter. mCherry and GFP signals were measured by flow cytometry following different durations of serum and amino acid starvation. Representative flow plots of live, single cells showing mCherry and GFP signal per genotype with 0 and 8 hours of amino acid and serum starvation.
(**D**) Representative histograms of mCherry/GFP signal ratio per genotype with 0 and 8 hours of starvation, cells are gated for live, single cells.
(**E**) Line plots of normalized mCherry/GFP signal in B16-F10 Cas9-expressing cells stably expressing the LC3-mCherry-GFP autophagy reporter treated with the indicated pgRNAs and increasing time under starvation conditions. Each point is the mean of n = 3 technical replicates +/- SEM, *P* value from two-sided Student's *t*-test (exact *P* values at times 0, 2, 4, 8 hrs are 0.006, 7.39^{-6}, 0.001, and 2.5^{-4}, respectively).

Raw gel images

Figure 4E

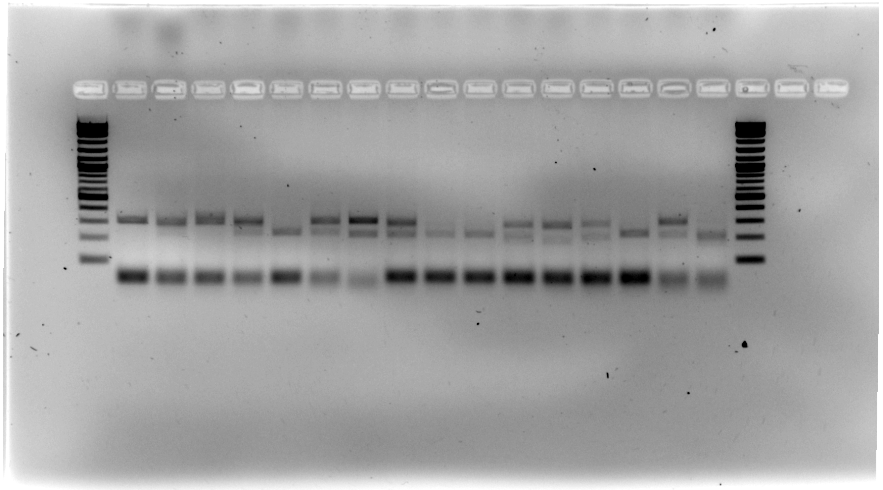

Figure 4F

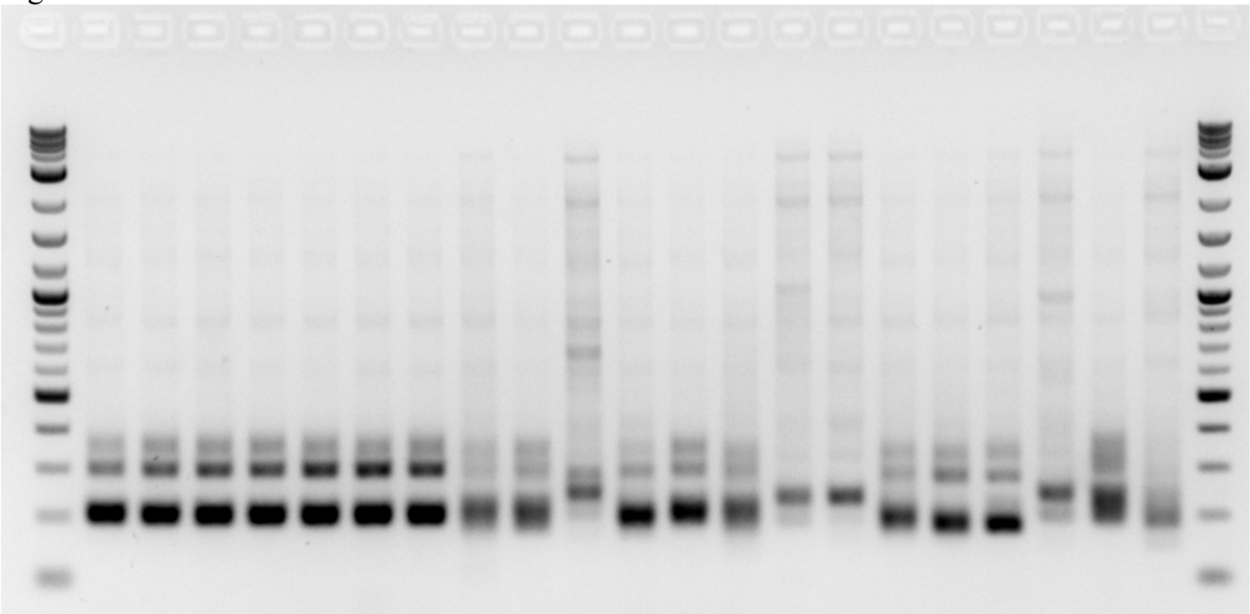

Figure 10A

Red = anti-Atg7, Green = anti alpha-Tubulin

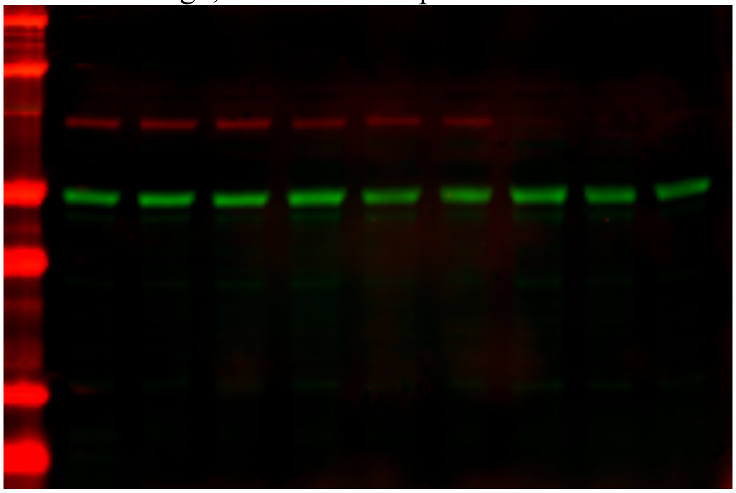

Red = anti-p62

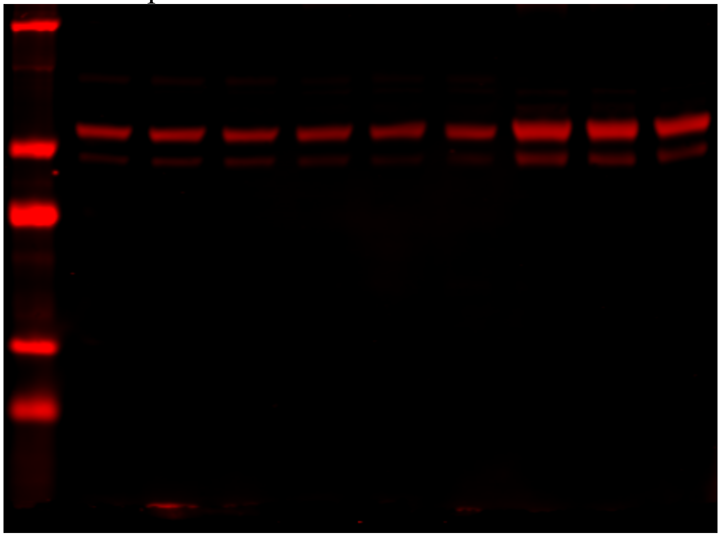

Supplement: Supplementary file 1 — Supplementary Information [file 41467_2024_44931_MOESM1_ESM.pdf]
