# Supplementary material for: Multiplexed screening reveals how cancer-specific alternative polyadenylation shapes tumor growth in vivo
Source: Nat Commun. 2024 Feb 1;15:959. doi: 10.1038/s41467-024-44931-x (PMC10834521; doi:10.1038/s41467-024-44931-x)
Supplement: Supplementary file 8 — Reporting Summary [file 41467_2024_44931_MOESM8_ESM.pdf]

nature portfolio

Corresponding author(s): Robert K. Bradley

Last updated by author(s): Dec 7, 2023

Reporting Summary

Nature Portfolio wishes to improve the reproducibility of the work that we publish. This form provides structure for consistency and transparency in reporting. For further information on Nature Portfolio policies, see our Editorial Policies and the Editorial Policy Checklist.

Statistics

For all statistical analyses, confirm that the following items are present in the figure legend, table legend, main text, or Methods section.

n/a	Confirmed	
☐	☒	The exact sample size (*n*) for each experimental group/condition, given as a discrete number and unit of measurement
☐	☒	A statement on whether measurements were taken from distinct samples or whether the same sample was measured repeatedly
☐	☒	The statistical test(s) used AND whether they are one- or two-sided<br>*Only common tests should be described solely by name; describe more complex techniques in the Methods section.*
☐	☒	A description of all covariates tested
☐	☒	A description of any assumptions or corrections, such as tests of normality and adjustment for multiple comparisons
☐	☒	A full description of the statistical parameters including central tendency (e.g. means) or other basic estimates (e.g. regression coefficient) AND variation (e.g. standard deviation) or associated estimates of uncertainty (e.g. confidence intervals)
☐	☒	For null hypothesis testing, the test statistic (e.g. *F*, *t*, *r*) with confidence intervals, effect sizes, degrees of freedom and *P* value noted<br>*Give P values as exact values whenever suitable.*
☐	☒	For Bayesian analysis, information on the choice of priors and Markov chain Monte Carlo settings
☐	☒	For hierarchical and complex designs, identification of the appropriate level for tests and full reporting of outcomes
☐	☒	Estimates of effect sizes (e.g. Cohen's *d*, Pearson's *r*), indicating how they were calculated

Our web collection on statistics for biologists contains articles on many of the points above.

Software and code

Policy information about availability of computer code

Data collection	No software was used.
Data analysis	Publicly available software was used in this study. Specific programs are RSEM (v1.2.4), Bowtie (v1.0.0), TopHat (v2.0.8b), MISO (v2.0), and Bioconductor (v3.7) within the R (v3.5.1) programming environment. FIJI/ImageJ (v2.0.0) and HALO (v2.0, Indica Labs) were used for image analysis. MAFFT (v7.0) was used for Sanger sequence alignments. No custom code was utilized for this study.

For manuscripts utilizing custom algorithms or software that are central to the research but not yet described in published literature, software must be made available to editors and reviewers. We strongly encourage code deposition in a community repository (e.g. GitHub). See the Nature Portfolio guidelines for submitting code & software for further information.

Data

Policy information about availability of data

All manuscripts must include a data availability statement. This statement should provide the following information, where applicable:

- Accession codes, unique identifiers, or web links for publicly available datasets
- A description of any restrictions on data availability
- For clinical datasets or third party data, please ensure that the statement adheres to our policy

Our proximal-Poly(A) KO (pKO) library has been deposited with Addgene (Pooled Library #81543 [https://www.addgene.org/Robert_Bradley/]). RNA-seq data generated as part of this study has been deposited in the Gene Expression Omnibus (accession number GSE212278 [https://www.ncbi.nlm.nih.gov/geo/query/

acc.cgi?acc=GSE212278]). Gene-level 3′ UTR measurements for all TCGA samples were downloaded from http://tc3a.org (51). Source data are provided with this paper. Data for Figures 1-2, 4-5 and Supplementary Figures 1-3, 5, 7-8 are included in Supplementary Data 1-5 and the Source Data file. All remaining data can be found in the Article, Supplementary and Source Data files.

Research involving human participants, their data, or biological material

Policy information about studies with human participants or human data. See also policy information about sex, gender (identity/presentation), and sexual orientation and race, ethnicity and racism.

Reporting on sex and gender	N/A
Reporting on race, ethnicity, or other socially relevant groupings	N/A
Population characteristics	N/A
Recruitment	N/A
Ethics oversight	N/A

Note that full information on the approval of the study protocol must also be provided in the manuscript.

Field-specific reporting

Please select the one below that is the best fit for your research. If you are not sure, read the appropriate sections before making your selection.

- [x] Life sciences
- [ ] Behavioural & social sciences
- [ ] Ecological, evolutionary & environmental sciences

For a reference copy of the document with all sections, see nature.com/documents/nr-reporting-summary-flat.pdf

Life sciences study design

All studies must disclose on these points even when the disclosure is negative.

Sample size	Samples sizes for pooled screens were based on results from our pilot screen as well as information garnered from published literature (Doench 2018, Chen et al 2015).
Data exclusions	No data were excluded.
Replication	All attempts at replication with both technical and biological replicates were successful. As described and shown in figure legends, most molecular assays were completed in biological triplicate (RT-PCR, Western blotting, qPCR). Cell growth assays were completed in biological triplicate and technical triplicate each time, and was performed by 2 different researchers to ensure reproducibility. Mouse experiments were done with 12 tumors per genotype tested. In vitro and in vivo screens were performed with 8 technical replicates.
Randomization	Individual animals were randomly assigned to be injected with B16-F10 cells treated with control or Atg7 pKO.
Blinding	For in vivo validation experiments, all researchers involved in tumor measuring and determination of individual animal endpoints were blinded from the genotype of the tumors to prevent bias.

Reporting for specific materials, systems and methods

We require information from authors about some types of materials, experimental systems and methods used in many studies. Here, indicate whether each material, system or method listed is relevant to your study. If you are not sure if a list item applies to your research, read the appropriate section before selecting a response.

Materials & experimental systems

n/a	Involved in the study	
[ ]	[x]	Antibodies
[ ]	[x]	Eukaryotic cell lines
[x]	[ ]	Palaeontology and archaeology
[ ]	[x]	Animals and other organisms
[x]	[ ]	Clinical data
[x]	[ ]	Dual use research of concern
[x]	[ ]	Plants

Methods

n/a	Involved in the study	
[x]	[ ]	ChIP-seq
[ ]	[x]	Flow cytometry
[x]	[ ]	MRI-based neuroimaging

Antibodies

Antibodies used	Antibodies used for western blotting at the following concentrations were Atg7 (AbCam Ab133528, 1:1000), Egln1 (Cell-Signaling Technology D31E11, 1:1000), p62/Sqstm1 (AbCam Ab109012, 1:1000), and Alpha-tubulin (Sigma-Aldrich Clone DM1, 1:2000) and all probed with secondary antibodies Anti-mouse or anti-rabbit IRDye (LI-COR Biosciences). Mouse Ki-67 (CST Clone 12202 1:2000 dilution) was utilized for immunohistochemical studies.
Validation	Atg7 (AbCam Ab133528, 1:1000) antibody confirmed for western blot analysis on mouse kidney lysate producing a single band of the expected protein size (https://www.abcam.com/atg7-antibody-epr6251-ab133528.html#lb).<br><br>Egln1 (Cell-Signaling Technology D31E11, 1:1000) antibody confirmed for western blot analysis on mouse fibroblast cell line (NIH/3T3) producing a single band of the expected protein size (https://www.cellsignal.com/products/primary-antibodies/phd-2-egln1-d31e11-rabbit-mab/4835).<br><br>p62/Sqstm1 (AbCam Ab109012, 1:1000) antibody confirmed for western blot analysis on mouse brain, heart and lung lysates producing a double band of the expected protein sizes (https://www.abcam.com/sqstm1--p62-antibody-epr4844-autophagosome-marker-ab109012.html#lb).<br><br>Alpha-tubulin (Sigma-Aldrich Clone DM1, 1:2000) antibody confirmed for western blot analysis on mouse fibroblast cell line (NIH/3T3) producing a single band of the expected protein size (https://www.thermofisher.com/antibody/product/alpha-Tubulin-Antibody-clone-DM1A-Monoclonal/62204).<br><br>Mouse Ki-67 (CST Clone 12202 1:2000 dilution) antibody confirmed for IHC using mouse colon , spleen and heart demonstrating the expected nuclear staining profiles reflecting known division rates of respective tissues (https://www.cellsignal.com/product/productDetail.jsp?productId=12202&utm_medium=b2b&utm_campaign=general).

Eukaryotic cell lines

Policy information about cell lines and Sex and Gender in Research

Cell line source(s)	B16-F10 cells were obtained from ATCC (CRL-6475) and cultured per the manufacturer's instructions. The Melan-A cell line was obtained from the Wellcome Trust Functional Genomics Cell Bank (original source of the cell line) and cultured per their instructions (Bennett et al., 1987).
Authentication	Cell lines were authenticated via RNA-seq expression profiling. B16-F10 cells were also spot checked as they express melanin and cell pellets are black (no other cell lines in use are black when pelleted).
Mycoplasma contamination	Cell lines tested negative for mycoplasma contamination.
Commonly misidentified lines (See ICLAC register)	None of the cell lines are commonly misidentified cell lines.

Animals and other research organisms

Policy information about studies involving animals; ARRIVE guidelines recommended for reporting animal research, and Sex and Gender in Research

Laboratory animals	Species: Mus musculus. Strain: C57BL/6. Sex: male. Age: 8 to 12 weeks. Animals were housed with a 14-hour light/10-hour dark cycle, with temperatures of 65-75°F (~18-23°C) and 40-60% humidity.
Wild animals	No wild animals were used in this publication.
Reporting on sex	For all mouse experiments male mice were used, as such the mouse data only applies to male mice.
Field-collected samples	No field collected samples were used or analyzed in this publication.
Ethics oversight	All mouse experiments were completed under an approved IACUC protocol () which was reviewed an approved by the Fred Hutch Cancer Center IACUC review committee.

Note that full information on the approval of the study protocol must also be provided in the manuscript.

Plants

Seed stocks	N/A
Novel plant genotypes	N/A
Authentication	N/A

Flow Cytometry

Plots

Confirm that:

- [x] The axis labels state the marker and fluorochrome used (e.g. CD4-FITC).
- [x] The axis scales are clearly visible. Include numbers along axes only for bottom left plot of group (a 'group' is an analysis of identical markers).
- [x] All plots are contour plots with outliers or pseudocolor plots.
- [x] A numerical value for number of cells or percentage (with statistics) is provided.

Methodology

Sample preparation	All cells were washed 3x with PBS and then resuspended in 100 uL of PBS and stained with LIVE/DEAD Fixable Violet (Thermo Fischer L34955) stain per the manufacturer's instructions. Cells were then passed through a 40-micron filter to generate a single cell suspension and then run on a BD FACSCelesta. Single cells were gated for live cells (BV421) and then the individual GFP and mCherry signal were measured in the FITC and CF594 channels, respectively. Data were analyzed in FlowJo v10.
Instrument	BD FACSCelesta
Software	FlowJo v10
Cell population abundance	All flow cytometry was performed on cells grown in culture and as such all cells in the dish represent the cell population of interest.
Gating strategy	FSC/SSC sorting for main cell population appeared relative uniform for B16-F10 cells, followed by sorting for single cells using FSC/FSC-H to remove doublets. Cells were then selected for low staining intensity with LIVE/DEAD Fixable Violet (BV421). The remaining population constituted live, single cells and were used to measure GFP, mCherry or propidium ioding (PE) signal for the relevant experiments.

- [ ] Tick this box to confirm that a figure exemplifying the gating strategy is provided in the Supplementary Information.
