# Supplementary material for: Multiplexed screening reveals how cancer-specific alternative polyadenylation shapes tumor growth in vivo
Source: Nat Commun. 2024 Feb 1;15:959. doi: 10.1038/s41467-024-44931-x (PMC10834521; doi:10.1038/s41467-024-44931-x)
Supplement: Supplementary file 10 — Description of Additional Supplementary Files [file 41467_2024_44931_MOESM10_ESM.pdf]

**Title: Supplementary Data File 1.**
**Description: Hazard ratios for progression-free and overall survival for all 30 TCGA cancer subtypes.** For each subtype, the hazard ratio of long median 3′ UTR versus short median 3′ UTR is given. The table also includes the standard deviation of the hazard ratio, the *p* value, and the number of patients included in the study.

**Title: Supplementary Data File 2.**
**Description: Median 3′ UTR measurement for TCGA cutaneous melanoma computed via two distinct algorithms.** Median 3′ UTR measurement per sample in the TCGA cutaneous melanoma (SKCM) dataset computed using the DaPars algorithm (15) or the APAlyzer algorithm (23), with the standard deviation of that measurement and binned such that 1 corresponds to short, 2 corresponds to medium, and 3 corresponds to long. The final column "same" indicates whether the patient has been placed in the same bin by both algorithms.

**Title: Supplementary Data File 3.**
**Description: Differentially polyadenylated transcripts identified by Poly(A)-seq between Melan-A and B16-F10 cells.** Includes summary of detected events, raw read counts, and the $\log_2$ ratio of (distal/ proximal) per transcript. Analysis was completed in a stranded fashion, and the forward and reverse data is included.

**Title: Supplementary Data File 4.**
**Description: Proximal poly(A) site knock out library (pKO Library) summary statistics per paired-guide RNA generation.** Table includes guide RNA sequences, poly(A) site target, on-target efficiency, specificity and other relevant information, as well as the final oligo sequence input to order the library.

**Title: Supplementary Data File 5.**
**Description: pKO library *in vitro* and *in vivo* CRISPR-Cas9 screen data.** Summary statistic scores per poly(A) site target, including the mean of all pgRNAs targeted to a specific poly(A) site, mean value normalized to control pgRNAs, *p* value per target, and FDR per target for the *in vitro* and *in vivo* screens.

**Title: Supplementary Data File 6.**
**Description: Oligonucleotide sequences utilized in paper.** Table includes primer names, gene targets, sequences, and specific uses. Includes CRISPR sgRNA and pgRNA sequences not included in pKO library (Supplementary Data File 4).
